# Characterization of the Teleneurology Patients at the Hospital Las Higueras de Talcahuano—Chile

**DOI:** 10.3389/fneur.2020.595577

**Published:** 2020-11-19

**Authors:** Freddy Constanzo, Paula Aracena-Sherck, Lorena Peña, Mery Marrugo, Jonathan Gonzalez, Gerardo Vergara, Cristóbal Alvarado

**Affiliations:** ^1^Neurology Unit, Hospital Las Higueras, Talcahuano, Chile; ^2^Medical Program in Adult Neurology, School of Medicine, Universidad Católica de la Santísima Concepción, Concepción, Chile; ^3^Department of Science, School of Medicine and Science, Universidad San Sebastián, Concepción, Chile; ^4^Unit of Teleprocesses, Hospital Las Higueras, Talcahuano, Chile; ^5^Department of Basic Sciences, School of Medicine, Universidad Católica de la Santísima Concepción, Concepción, Chile

**Keywords:** telemedicine, teleneurology, adult neurology, pathologies, waiting-list

## Abstract

**Background:** Chile has a shortage of medical experts, including neurologists. The remote neurology program at Las Higueras Hospital in Talcahuano (HHT) was implemented in 2015 to decrease the number of patients waiting for their first appointment.

**Methods:** This retrospective study analyzed a cohort of 2,904 ambulatory patients evaluated in the teleneurology program at the HHT between 2015 and 2019 who were referred from 16 primary and 3 tertiary healthcare centers.

**Results:** Out of the 2,904 patients included in the study, 1,020 patients (35%) were male, and 1,884 (65%) were female. In total, 1,346 (46.0%) patients were under 60 years old (408 male and 938 female), and 1,558 (54%) were over 60 years old (612 male and 946 female). The patients were referred to a neurologist in the teleneurology program from different primary healthcare centers (93.5%) and tertiary healthcare centers (6.5%). The most common diseases diagnosed through teleneurology were, in decreasing order, headache (29.4%), Alzheimer's disease and other dementias (15.9%), and epilepsy (11.4%). From July 2018, we analyzed the patients' destination after the first teleneurology consultation. In the cohort of 634 patients who had their first consultation via the teleneurology program, 547 (86.3%) were instructed to continue follow-up via telemedicine.

**Conclusions:** Data from this study show, for the first time in Chile, the significant contribution of the teleneurology program at the HHT to the diagnosis of a broad range of diseases in a substantial number of patients referred from primary and tertiary healthcare centers.

## Background

Chile is a South American country with 19,458,310 inhabitants as of 2020 (1), with a lack of medical specialists. Most of the specialists work in the tertiary healthcare system, which is concentrated in large cities (2, 3). Currently, Chile has 605 licensed neurologist (4), but only 292 neurologists currently work in the public healthcare system (4, 5), which provides medical care to ~80% of the total population (6). Hence, the number of patients waiting for their first appointment increases every year, and neurology ranks seventh among Chilean medical specialties; as of December 2019, there were 57,837 patients waiting for a first appointment with a neurologist (7). The lack of timely access to medical services is a growing national problem, especially among people with disabilities such as those with neurological diseases (7, 8). The shortage of medical specialists has exacerbated the country's geographical difficulties in delivering tertiary healthcare; however, this situation provides an opportunity for telemedicine to improve patient access to professional medical services. Therefore, the implementation and the development of telemedicine in the Chilean public healthcare system is a good means of overcoming the shortage of experts and the transportation problems for ambulatory patients (9) and acute stroke patients (10).

The Talcahuano Health Service started the teleneurology program in March 2015 to help manage adult neurological patients waiting for their first consultation (9). The program runs in a synchronous mode, in which the ambulatory patient consults a general practitioner, who has been trained for this purpose in a local medical institution and connects the patient with a neurologist at the Hospital Las Higueras de Talcahuano (HHT) via an HDTV video conference system (11). The teleneurology program allows the remote care of patients at local primary and secondary medical institutions and has been shown to improve access to medical care from neurologists, with high levels of user satisfaction (11–13). Furthermore, the teleneurology program has also produced very good results with regard to reducing the waiting list for a first consultation, providing follow-up visits for patients with adequate control of their condition (9) and maintaining access to services during the coronavirus disease 2019 pandemic (14–16).

The burden of neurological disease is similar among different countries, with some differences depending mainly on the level of development, and it is measured annually (17–19). The primary neurological diseases are motor neuron diseases, multiple sclerosis, tetanus, other neurological disorders, Parkinson's disease, encephalitis, tension-type headache, brain and other central nervous system (CNS) cancers, traumatic brain injury, spinal cord injury, epilepsy, meningitis, Alzheimer's disease and other dementias, migraine, and stroke. Therefore, it is necessary to reorganize the different levels of the healthcare system to face these challenges. The systematic use of teleneurology has been shown to be effective for many neurological disorders (9, 20, 21). Recent studies have provided a detailed description of the types of pathologies that can be efficiently treated through telemedicine (20–22); however, in Chile, there are no published reports that describe the most common neurological pathologies that could be treated by teleneurology in outpatient clinics. In this study, we performed a retrospective analysis of 2,904 patients who were examined, diagnosed, and treated between 2015 and 2019 in the teleneurology program at HHT. In addition, we classified the patients by sex, age, place of origin, and diagnosis, with the aim of demonstrating that teleneurology manages to address the majority of diseases contributing to the ambulatory neurological disease burden in Chile. This manuscript, for the first time in Chile, details the most common pathologies in ambulatory patients in Chile that can be managed by teleneurology.

## Methods

### Description of the Study Population

This retrospective cohort study analyzed the data of patients who accessed the teleneurology program between June 2015 and October 2019. All the patients included in this study had their first appointment with the neurologist by telepresence (synchronous clinic videoconference); the details are described in Constanzo et al. (11). The patients included in this study were referred from the following primary health departments that depend on the HHT: (i) community family health centers (CECOSF for the Spanish acronym): CECOSF 8 mayo, CECOSF Esmeralda, CECOSF Leocán Portus, CECOSF Libertad Gaete, and CECOSF Los Lobos—La Gloria; (ii) family health centers (CESFAM for the Spanish acronym): CESAFM Paulina Avendaño Pereda, CESFAM Bellavista, CESFAM Dr. Alberto Reyes, CESFAM Hualpencillo, CESFAM La Floresta, CESFAM Lirquén, CESFAM Los Cerros, CESFAM Penco, CESFAM San Vicente, CESFAM Talcahuano Sur, CESFAM Alcalde Leocán Portus, and the Community Dementia Centre (CDC) Kelluwün; and (iii) tertiary healthcare centers: HHT, Tomé Hospital, and Penco-Lirquén Hospital.

### Teleneurology System

The teleneurology program implemented at the HHT combines the simultaneous live care by a general practitioner *in situ* and the remote primary care offered by a neurology specialist in specifically designed HHT facilities. For each video session in the teleprocess unit (synchronous), a referral and a reference web platform are used, which is integrated in the hospital electronic clinical record with an HL7 standard and has a Secure Sockets Layer security server for encryption. The inclusion and the exclusion criteria for the patients in this study were previously described in Constanzo et al. (11) (Supplementary Table 1). During this study, the HHT Neurology Unit consisted of 11 neurologists and two general practitioners. The neurologist group dedicated, as a group, 74 h per week to consultation *in situ* at HHT and 17 h per week to the teleneurology program. The general practitioners dedicated, as a group, 21 h per week for (i) coordination of care and follow-up of patients waiting for an appointment, (ii) extension of chronic patient prescriptions, and (iii) follow-up of patients with a prolonged medical license or in the process of requesting a disability pension (9). This allows the team of neurologists to focus exclusively on the resolution of the patients' neurological problems. Finally, all participant nneurologists receive training in teleneurology by the Teleneurology Unit at the HHT in association with the Universidad Católica de la Santísima Concepción, Chile.

### Neurological Diseases Attended in the Teleneurology Program

The teleneurology patients were categorized as follows: (i) headache: chronic migraine, migraine, tension-type headache, chronic tension-type headache, and other headaches; (ii) Alzheimer's disease and other dementias: Alzheimer's disease, dementia syndrome (first evaluation), mild cognitive impairment, vascular and Alzheimer's dementia, vascular dementia, Lewy body dementia, traumatic brain injury dementia, and other dementias; (iii) epilepsy; (iv) other neurological disorders: brain and other CNS cancer, vertigo, motor mononeuropathy (ocular and facial), intellectual disability, hemifacial spasm, tics and dystonia, ataxias, sleep disorders, motor neuron disease, dystrophies and myotonias, myasthenia gravis, and others; (v) Parkinson's disease and parkinsonism: Parkinson's disease, parkinsonian syndrome (first evaluation), atypical parkinsonian disorders; (vi) neuropathies and neuropathic pain: polyneuropathy, radiculopathy, trigeminal neuralgia, postherpetic neuralgia, and others; (vii) other non-neurological disorders: syncope, other psychiatric disorders; depression; fibromyalgia, and others; (viii) essential tremor; (ix) stroke: ischemic, hemorrhagic, transient ischemic attack (TIA), others; (x) traumatic brain injury; and (xi) spinal cord injury.

### Statistical Analysis

The descriptive analysis of patient data is reported here as the frequencies and the percentages for continuous and categorical variables. All analyses were performed with SPSS, version 25.

## Results

### Patient Cohort Description

**Figure 1:**
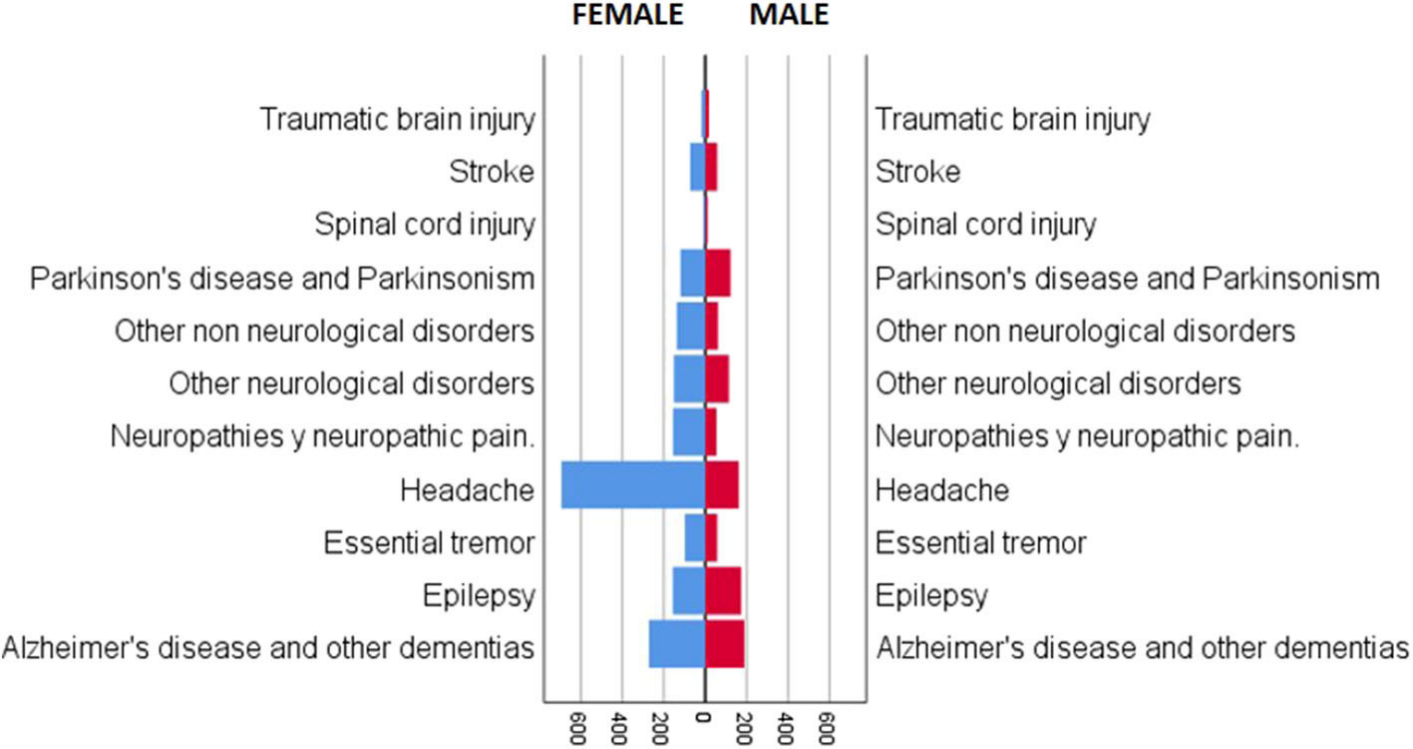
Patients categorized by gender (female and male) in the teleneurology program at the Hospital las Higueras of Talcahuano.

This retrospective cohort study included 2,904 patients who were evaluated in the teleneurology program between June 2015 and October 2019. In summary, 1,020 patients (35%) were male, and 1,884 (65%) were female (Figure 1, Table 1); 1,346 (46.0%) patients were under 60 years old (408 male and 938 female), and 1,558 (54%) were over 60 years old (612 male and 946 female) (Table 1). During the study period, we performed a total of 5,874 telepresence appointments; 2,904 (49%) were first appointments, and 2,970 (51%) were follow-up visits.

**Table 1:** Patients categorized by age (under 60 and over 60), and place of origin in the teleneurology program at the Hospital las Higueras of Talcahuano.

	**Female**	**%**	**Male**	**%**	**Total (*n* = 2,904)**	**%**
**Age**						
Total	1,884	65%	1,020	35%	2,904	100.0%
Under 60 years	938	32.3%	408	14.0%	1,346	46.3%
Over 60 years	946	32.6%	612	21.1%	1,558	53.7%
**Place of origin**						
CDC Kelluwün	35	1.2%	29	1.0%	64	2.2%
CECOSF 8 mayo	9	0.5%	2	0.1%	11	0.4%
CECOSF Esmeralda	18	1.0%	10	0.3%	28	1.0%
CECOSF Libertad Gaete	1	0.1%	0	0.0%	1	0.0%
CECOSF Los Lobos—La Gloria	2	0.1%	0	0.0%	2	0.1%
CESFAM Paulina Avendaño Pereda	283	15.0%	162	5.6%	445	15.3%
CESFAM Bellavista	36	1.9%	15	0.5%	51	1.8%
CESFAM Dr. Alberto Reyes	108	5.7%	50	1.7%	158	5.4%
CESFAM Hualpencillo	201	10.7%	116	4.0%	317	10.9%
CESFAM La Floresta	178	9.4%	74	2.5%	252	8.7%
CESFAM Lirquén	23	1.2%	22	0.8%	45	1.5%
CESFAM Los Cerros	139	7.4%	74	2.5%	213	7.3%
CESFAM Penco	133	7.1%	58	2.0%	191	6.6%
CESFAM San Vicente	216	11.5%	102	3.5%	318	11.0%
CESFAM Talcahuano Sur	169	9.0%	93	3.2%	262	9.0%
CESFAM Alcalde Leocán Portus	234	12.4%	123	4.2%	357	12.3%
Hospital de Tomé	69	3.7%	56	1.9%	125	4.3%
Hospital Las Higueras	6	0.3%	4	0.1%	10	0.3%
Hospital Penco Lirquén	24	1.3%	30	1.0%	54	1.9%
Total	1,884	100.0%	1,020	35.1%	2,904	100%

**Figure 2:**
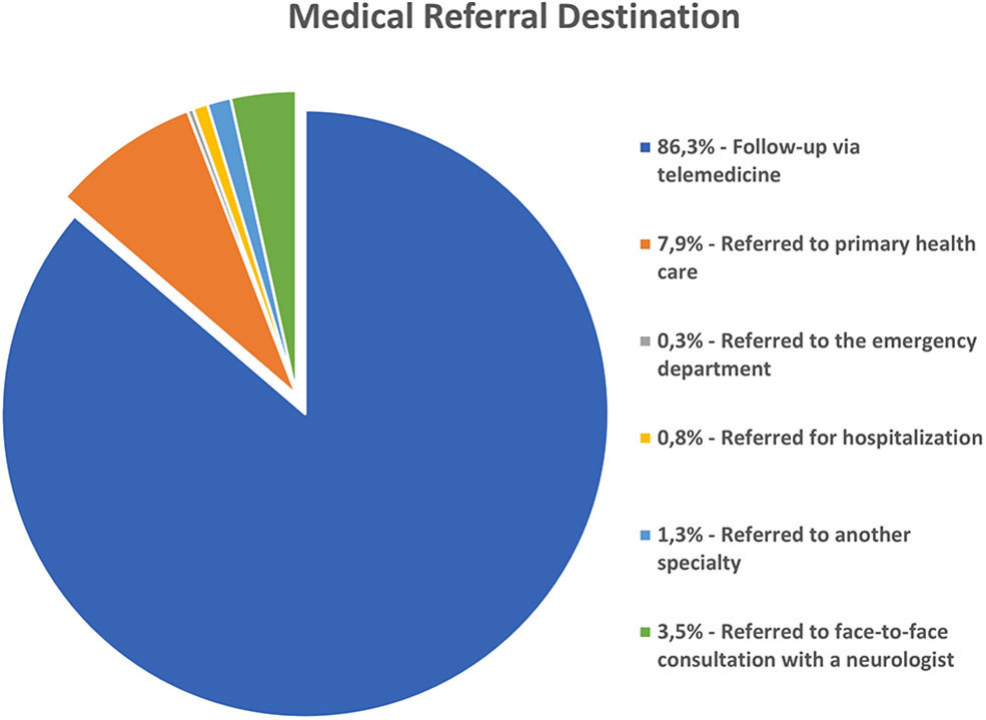
Medical referral destination from a cohort of 634 first consultation patients.

Interestingly, after July 2018, we were able to determine the medical referral destination of the patients after the first consultation. From a cohort of 634 first consultation patients, 547 (86.3%) patients were instructed to continue follow-up via telemedicine, 50 (7.9%) patients were referred to primary healthcare, and 37 (5.8%) patients, given their complexity, were referred to the tertiary level of healthcare as follows: two (0.3%) patients to the emergency department, five (0.8%) patients for hospitalization, eight (1.3%) patients to another specialty, and 22 (3.5%) patients to face-to-face consultation with a neurologist (Figure 2).

### Health Centers Associated With the Teleneurology Program

The patients were referred to a neurologist by telepresence from different primary healthcare centers (93.5%) as follows (Table 1): community family health centers: CDC Kelluwün (2.2%), CECOSF 8 mayo (0.4%), CECOSF Esmeralda (1.0%), CECOSF Leocán Portus (0.5%), CECOSF Libertad Gaete (0.0%), CECOSF Los Lobos—La Gloria (0.1%); family health centers: CESFAM Paulina Avendaño Pereda (15.3%), CESFAM Bellavista (1.8%), CESFAM Dr. Alberto Reyes (5.4%), CESFAM Hualpencillo (10.9%), CESFAM La Floresta (8.7%), CESFAM Lirquén (1.5%), CESFAM Los Cerros (7.3%), CESFAM Penco (6.6%), CESFAM San Vicente (11.0%), CESFAM Talcahuano Sur (9.0%), and CESFAM Alcalde Leocán Portus (11.8%); and tertiary healthcare centers (6.5%): Tomé Hospital (4.3%), HHT (0.3%), and Penco-Lirquén Hospital (1.9%) (Table 1).

### Characterization of Neurological Diseases Attended in the Teleneurology Program

The patients were stratified by pathology, from the most common to the least as follows: (i) headache −855 (29%): chronic migraine −309 (10.6%), migraine −253 (8.7%), tension-type headache −146 (5%), chronic tension-type headache −131 (4.5%), and other headaches −16 (0.6%); (ii) Alzheimer's disease and other dementias −461 (15.9%): Alzheimer's disease −227 (7.8%), dementia syndrome (first evaluation) −79 (2.7%), mild cognitive impairment −72 (2.5%), vascular and Alzheimer's dementia −30 (1%), other dementias −29 (1%), vascular dementia −14 (0.5%), Lewy body dementia—seven (0.2%), traumatic brain injury dementia—three (0.1%); (iii) epilepsy −331 (11.4%); (iv) other neurological disorders −265 (9.1%): others −62 (2.1%), brain and other CNS cancer −47 (1.6%), vertigo −40 (1.4%), motor mononeuropathy (ocular and facial) −25 (0.9%), intellectual disability −23 (0.8%), hemifacial spasm, tics, and dystonia −18 (0.6%), ataxias −12 (0.4%), sleep disorders −12 (0.4%), motor neuron disease −10 (0.3%), dystrophies and myotonias—eight (0.3%), myasthenia gravis—eight (0.3%); (v) Parkinson's disease and parkinsonism −242 (8.3%): Parkinson's disease −177 (6.1%), parkinsonian syndrome (first evaluation) −55 (1.9%), atypical parkinsonian disorders −10 (0.3%); (vi) neuropathies and neuropathic pain −210 (7.2%): polyneuropathy −77 (2.7%), radiculopathy −42 (1.4%), others −25 (0.9%), trigeminal neuralgia −23 (0.8%), postherpetic neuralgia −17 (0.6%); (vii) other non-neurological disorders −199 (6.9%): others −93 (3.2%), syncope −39 (1.3%), other psychiatric disorders −34 (1.2%), depression −26 (0.9%), fibromyalgia—seven (0.2%); (viii) essential tremor −156 (5.4%); (ix) stroke −130 (4.5%): ischemic −93 (3.2%), hemorrhagic −23 (0.8%), TIA −13 (0.4%), others—one (~0%); (x) traumatic brain injury −36 (1.2%); and (xi) spinal cord injury −19 (0.7%) (Table 2).

**Table 2:** Patients categorized by neurological pathology in the teleneurology program at the Hospital las Higueras of Talcahuano.

	**Female**	**%**	**Male**	**%**	**Total**	**%**
Headache	694	23.9%	161	5.5%	855	29.4%
Chronic migraine	266	9.2%	43	1.5%	309	10.6%
Migraine	216	7.4%	37	1.3%	253	8.7%
Tension-type headache	101	3.5%	45	1.5%	146	5.0%
Chronic tension-type headache	100	3.4%	31	1.1%	131	4.5%
Other headaches	11	0.4%	5	0.2%	16	0.6%
Alzheimer's disease and other dementias	272	9.4%	189	6.5%	461	15.9%
Alzheimer's disease	154	5.3%	73	2.5%	227	7.8%
Dementia syndrome (first evaluation)	32	1.1%	47	1.6%	79	2.7%
Mild cognitive impairment	40	1.4%	32	1.1%	72	2.5%
Vascular and Alzheimer's dementia	13	0.4%	17	0.6%	30	1.0%
Other dementias	17	0.6%	12	0.4%	29	1.0%
Vascular dementia	13	0.4%	1	0.0%	14	0.5%
Lewy body dementia	3	0.1%	4	0.1%	7	0.2%
Traumatic brain injury dementia	0	0.0%	3	0.1%	3	0.1%
Epilepsy	157	5.4%	174	6.0%	331	11.4%
Other neurological disorders	151	5.2%	114	3.9%	265	9.1%
Others	35	1.2%	27	0.9%	62	2.1%
Brain and other central nervous system cancer	29	1.0%	18	0.6%	47	1.6%
Vertigo	28	1.0%	12	0.4%	40	1.4%
Motor mononeuropathy (ocular and facial)	16	0.6%	9	0.3%	25	0.9%
Intellectual disability	6	0.2%	17	0.6%	23	0.8%
Hemifacial spasm, tics, and dystonia	12	0.4%	6	0.2%	18	0.6%
Ataxias	5	0.2%	7	0.2%	12	0.4%
Sleep disorders	5	0.2%	7	0.2%	12	0.4%
Motor neuron disease	2	0.1%	8	0.3%	10	0.3%
Dystrophies and myotonias	7	0.2%	1	0.0%	8	0.3%
Myasthenia gravis	6	0.2%	2	0.1%	8	0.3%
Parkinson's disease and parkinsonism	120	4.1%	122	4.2%	242	8.3%
Parkinson's disease	85	2.9%	92	3.2%	177	6.1%
Parkinsonian syndrome (first evaluation)	32	1.1%	23	0.8%	55	1.9%
Atypical parkinsonian disorders	3	0.1%	7	0.2%	10	0.3%
Neuropathies and neuropathic pain	156	5.4%	54	1.9%	210	7.2%
Polyneuropathy	50	1.7%	27	0.9%	77	2.7%
Radiculopathy	29	1.0%	13	0.4%	42	1.4%
Others	22	0.8%	3	0.1%	25	0.9%
Trigeminal neuralgia	22	0.8%	1	0.0%	23	0.8%
Postherpetic neuralgia	10	0.3%	7	0.2%	17	0.6%
Other non-neurological disorders	138	4.8%	61	2.1%	199	6.9%
Others	67	2.3%	26	0.9%	93	3.2%
Syncope	24	0.8%	15	0.5%	39	1.3%
Other psychiatric disorders	20	0.7%	14	0.5%	34	1.2%
Depression	20	0.7%	6	0.2%	26	0.9%
Fibromyalgia	7	0.2%	0	0.0%	7	0.2%
Essential tremor	98	3.4%	58	2.0%	156	5.4%
Stroke	72	2.5%	58	2.0%	130	4.5%
Ischemic	49	1.7%	44	1.5%	93	3.2%
Hemorrhagic	16	0.6%	7	0.2%	23	0.8%
Transient ischemic attack	7	0.2%	6	0.2%	13	0.4%
Others	0	0.0%	1	0.0%	1	0.0%
Traumatic brain injury	18	0.6%	18	0.6%	36	1.2%
Spinal cord injury	8	0.3%	11	0.4%	19	0.7%

### Characterization of Neurological Diseases by Age Attended in the Teleneurology Program

**Figure 3:**
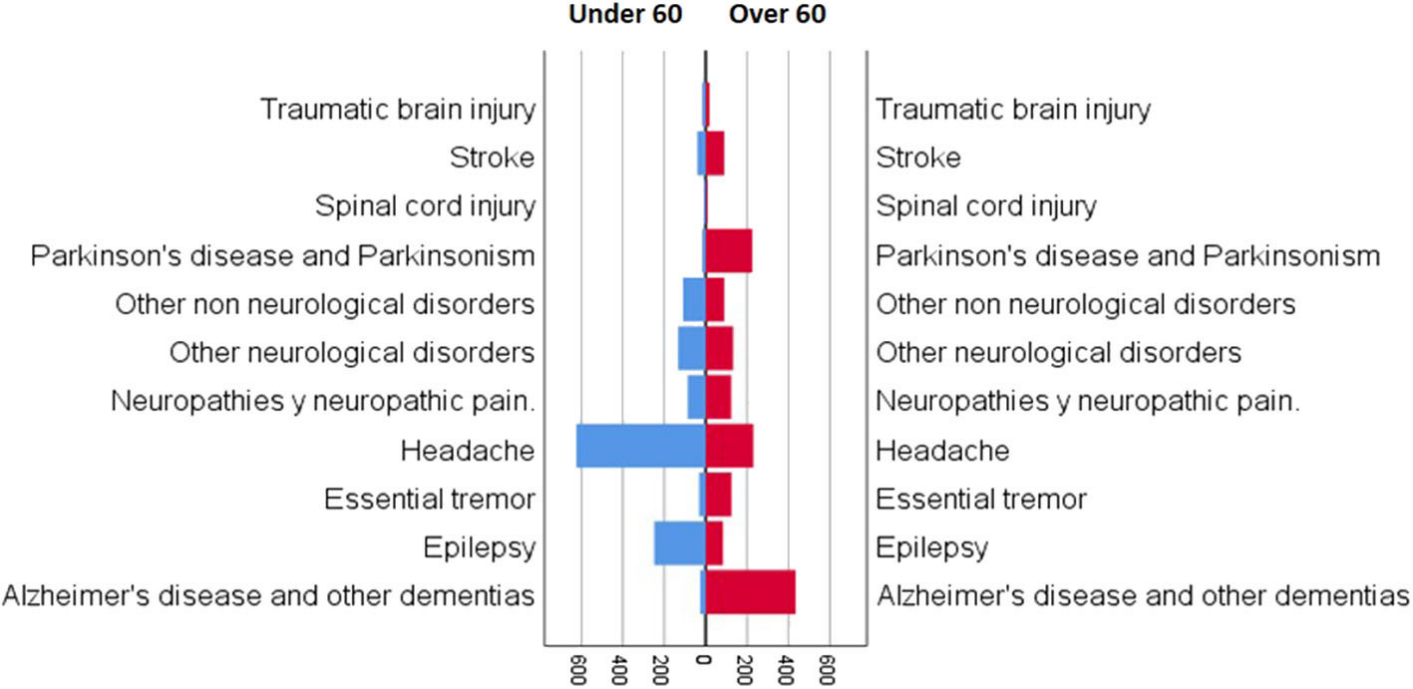
Patients categorized by age (under 60 and over 60) and neurological pathology in the teleneurology program at the Hospital las Higueras of Talcahuano.

The patients were stratified by age and pathology, from the most common to the least as follows (Figure 3): under 60 years old: (i) headache −625 (46%), (ii) epilepsy −248 (18%), (iii) other neurological disorders −133 (10%), (iv) other non-neurological disorders −110 (8.2%), (v) neuropathies and neuropathic pain −87 (6.5%), (vi) stroke −41 (3.0%), (vii) essential tremor −32 (2.4%), (viii) Alzheimer's disease and other dementias −26 (1.9%), (ix) Parkinson's disease and parkinsonism −17 (1.3%), (x) traumatic brain injury −17 (1.3%), and (xi) spinal cord injury −10 (0.7%) (Table 3); and over 60 years old: (i) Alzheimer's disease and other dementias −435 (28%), (ii) headache −230 (15%), (iii) Parkinson's disease and parkinsonism −225 (14%), (iv) other neurological disorders −132 (8.5%), (v) essential tremor −124 (8.0%), (vi) neuropathies and neuropathic pain −123 (7.9%), (vii) other non-neurological disorders −89 (5.7%), (viii) stroke −89 (5.7%), (ix) epilepsy −83 (5.3%), (x) traumatic brain injury −19 (1.2%), and (xi) spinal cord injury—nine (0.6%) (Table 3).

**Table 3:** Patients categorized by age (under 60 and over 60) and neurological pathology in the teleneurology program at the Hospital las Higueras of Talcahuano.

	**Gender**				**Total**	
	**Female**	**%**	**Male**	**%**	***n***	**%**
**Under 60**						
Headache	524	39%	101	7.5%	625	46%
Epilepsy	121	9.0%	127	9.4%	248	18%
Other neurological disorders	75	5.6%	58	4.3%	133	10%
Other non-neurological disorders	78	5.8%	32	2.4%	110	8.2%
Neuropathies and neuropathic pain	71	5.3%	16	1.2%	87	6.5%
Stroke	23	1.7%	18	1.3%	41	3.0%
Essential tremor	18	1.3%	14	1.0%	32	2.4%
Alzheimer's disease and other dementias	14	1.0%	12	0.9%	26	1.9%
Parkinson's disease and parkinsonism	4	0.3%	13	1.0%	17	1.3%
Traumatic brain injury	6	0.4%	11	0.8%	17	1.3%
Spinal cord injury	4	0.3%	6	0.4%	10	0.7%
Total under 60	938	70%	408	30%	1,346	100%
**Over 60**						
Alzheimer's disease and other dementias	258	17%	177	11%	435	28%
Headache	170	11%	60	4%	230	15%
Parkinson's disease and parkinsonism	116	7,4%	109	7%	225	14%
Other neurological disorders	76	4,9%	56	4%	132	8.5%
Essential tremor	80	5,1%	44	3%	124	8.0%
Neuropathies and neuropathic pain	85	5,5%	38	2%	123	7.9%
Other non-neurological disorders	60	3,9%	29	2%	89	5.7%
Stroke	49	3,1%	40	3%	89	5.7%
Epilepsy	36	2,3%	47	3%	83	5.3%
Traumatic brain injury	12	0,8%	7	0.4%	19	1.2%
Spinal cord injury	4	0,3%	5	0.3%	9	0.6%
Total over 60	946	61%	612	39%	1,558	100%

## Discussion

Neurological disorders are recognized as a group of increasingly common diseases due to an increase in the life expectancy of the population and the current lifestyle (23). Neurological diseases impose a high disease burden on patients, their families, and society (17). In Chile, this problem is more acute than in developed countries due to the shortage of neurologists and the concentration of the existing neurologists in the country's large cities (3).

Telemedicine provides the opportunity to improve patient access to specialized medical care, especially for patients with mobility disorders and those who live far from tertiary healthcare centers. Furthermore, telemedicine has emerged as a tool that allows the provision of faster and more timely care to patients who are awaiting evaluation by a specialist (9). In January 2015, the HHT created a teleneurology program for its patients based out of the Neurology Department, with the aim of improving access to specialists through an appointment with a general practitioner at a local primary healthcare center with the synchronous telepresence of the neurologist located at HHT.

The present study aimed to characterize the population of patients seen between 2015 and 2019 according to the diagnosed pathology. During this period, a total of 2,904 patients were seen, with a total of 5,874 consultations by telepresence, which represents a national milestone for the volume of patients seen. The teleneurology program has covered, since its inception, a wide spectrum of neurological diagnoses, and the most common diagnoses are described in this study. The most common pathologies reported in this study are headache, Alzheimer's disease and other dementias, and epilepsy. However, these frequencies changed when the patients were categorized by age; as expected, patients over 60 years old showed an increasing prevalence in neurodegenerative diseases and stroke. These patients showed a greater cognitive and mobilization disability; therefore, they are patients who require greater healthcare, which is facilitated by teleneurology. The frequency of cases reported in the teleneurology program is similar to the observed global burden of neurological diseases (19). Interestingly, we observed a different number of patients between genders. This is likely to be rooted in cultural differences, regarding patient compliance of Chilean females and males, characteristic in developing countries (24).

This study analyzed the disease burden on outpatients and did not consider patients who receive care in an emergency setting and require hospitalization; hence, the teleneurology program does not report a high number of stroke patients because these patients are treated at the HHT. The stroke patients who were included in this study were medical referrals from the HHT. Usually, these patients benefit from the teleneurology program when they cannot attend the hospital for follow-up after discharge from HHT. Therefore, in our opinion, the implementation of similar teleneurology programs could be aimed at controlling the burden of ambulatory disease, and a different program might be required for the treatment of stroke in an emergency context, depending on the availability of health system resources, as previously described in Chile (10) and globally (25).

Previous studies have reported the benefits of teleneurology consultations in outpatient settings in first-world countries (20); however, our study for the first time provides relevant information on the pathologies that are possible to treat through a teleneurology network in a developing country. The report of this experience clearly presents an opportunity to achieve greater equity in the medical coverage needed for patients with neurological diseases in a country with an inadequate geographical distribution of neurologists. It is interesting to note that, as of 2018, the telepresence program followed 634 patients treated via the telepresence modality, demonstrating that most patients (574, 86.3%) benefit from maintaining control of their condition via the same telepresence system. Furthermore, it should be noted that only 37 patients (5.8%) were referred to the tertiary level, which agrees with previous reports (20).

The future challenge for our teleneurology team is to dedicate time and effort to increase awareness of the importance of telemedicine among healthcare workers. Additionally, special effort must be exerted to convince the authorities and private parties of the importance of this service to guarantee greater support from policy makers. Currently, we are working on diversifying teleneurology services, which include call centers, teleneurology at home, and an education platform open to the community, and we are also collaborating to create new telemedicine units for additional specialties at HHT.

## Conclusions

The implementation of teleneurology programs such as ours could solve similar problems of lack of access to specialists in different regions of our country and the world. Our work group believes that the teleneurology program adequately responds to most outpatient conditions that increase the burden of neurological disease. Through the teleneurology program, we can easily determine which patients can continue follow-up via telemedicine and which need to be referred to tertiary healthcare facilities.

## Data Availability Statement

The original contributions presented in the study are included in the article/Supplementary Material, further inquiries can be directed to the corresponding author/s.

## Ethics Statement

The studies involving human participants were reviewed and approved by Ethics Committee of the Talcahuano Health Service. Written informed consent for participation was not required for this study in accordance with the national legislation and the institutional requirements.

## Author Contributions

FC contributed to the study conception and design, patients' medical care, and manuscript writing. PA-S contributed to manuscript writing and English editing. LP, MM, and JG contributed to the patients' medical care. GV supervised the teleprocesses for the teleneurology program at HHT. CA contributed to the coordination of researchers, statistical analysis, manuscript writing, and English editing. All authors contributed to the article and approved the submitted version.

## Conflict of Interest

The authors declare that the research was conducted in the absence of any commercial or financial relationships that could be construed as a potential conflict of interest.

## Abbreviations

NU: Neurology Unit
HHT: Hospital las Higueras of Talcahuano (HHT)
SST: Health Service of Talcahuano of the Ministry of Health.
