# Supplementary material for: Characterization of the Teleneurology Patients at the Hospital Las Higueras de Talcahuano—Chile
Source: Front Neurol. 2020 Nov 19;11:595577. doi: 10.3389/fneur.2020.595577 (PMC7711163; doi:10.3389/fneur.2020.595577)
Supplement: Supplementary file 1 [file Data_Sheet_1.PDF]

SUPLEMENTARY TABLE 1: INCLUSION AND EXCLUSION CRITERIA FOR POTENTIAL PATIENTS OF THE TELENEUROLOGY UNIT OF THE HOSPITAL LAS HIGUERAS DE TALCAHUANO.

**Inclusion criteria:** According to the reference and counter-referral protocol of the Hospital Las Higueras de Talcahuano for primary health care. The patients are referred with a complete study available in primary health care or performed during previous in-patient admissions. All patient to be included in this study, must have at least one of the 15 below mentioned pathologies.	**Exclusion criteria:** According to the reference and counter-referral protocol of the Hospital Las Higueras de Talcahuano for primary health care. These patients must be immediately derived to the Emergency Room or the Neurology Clinic. According to severity.
1- Ischemic or recent hemorrhagic stroke: control and follow-up after hospital discharge.<br>2.- Chronic daily headache<br>3.- Non-refractory epilepsy<br>4.- Dementia syndrome: study phase<br>5.- Dementias<br>6.- Polyneuropathies. chronic phase<br>7.- Chronic movement disorder (eg: essential tremor. chorea. parkinsonian syndrome)<br>8.- Parkinson's disease	1.- Ischemic or hemorrhagic stroke in the acute phase<br>2.- Transient ischemic crisis<br>3.- Thunder or ictal headache<br>4.- Seizures of recent onset<br>5.- Convulsive status<br>6.- Refractory epilepsy<br>7.- Subacute or rapidly progressive dementia<br>8.- Delirium<br>9.- Polyneuropathies. acute polyradiculopathies<br>10.- Movement disorders of acute onset or extrapyramidal emergencies.

9.- Vertiginous syndrome in chronic phase. as second opinion after evaluation by otorhinolaryngology.<br><br>10. Post-traumatic brain injury syndrome (chronic phase).<br><br>11.- Peripheral facial paralysis: before diagnostic doubt. in patient without symptoms and / or associated focal focal signs.<br><br>12.- Neuropathic pain. chronic phase<br><br>13.- Neuropathic pain. acute phase. only if etiology is very evident and treatable in primary care (eg: post herpetic neuralgia)<br><br>14.- Myasthenia gravis: chronic control in controlled symptoms with therapy<br><br>15.- Acceptance of the patient to use this type of care	myoplegia. hemiplegia. paraplegia or imperfect motility.<br><br>11.- Acute vertiginous syndrome with neurological symptoms and warning signs<br><br>12.- Cranial brain trauma (acute phase)<br><br>13.- Facial paralysis: patient with symptoms and / or associated central focal signs.<br><br>14.- Myasthenic syndrome: differential diagnosis study in acute phase<br><br>15.- Myasthenia gravis with poor response to usual therapy.<br><br>16.- Demyelinating diseases<br><br>17.- Mental disability or minors (law 28.584 article 28)<br><br>18.- Patient refusal to use this type of care
